# Exploring the *in silico* adaptation of the Nephroblastoma Oncosimulator to MRI scans, treatment data, and histological profiles of patients from different risk groups

**DOI:** 10.3389/fphys.2025.1465631

**Published:** 2025-04-17

**Authors:** Marcel Meyerheim, Foteini Panagiotidou, Eleni Georgiadi, Dimitrios Soudris, Georgios Stamatakos, Norbert Graf

**Affiliations:** ^1^ Pediatric Oncology and Hematology, Faculty of Medicine, Saarland University, Homburg, Germany; ^2^ In Silico Oncology and In Silico Medicine Group, Institute of Communication and Computer Systems, School of Electrical and Computer Engineering, National Technical University of Athens, Athens, Greece; ^3^ Biomedical Engineering Department, University of West Attica, Athens, Greece

**Keywords:** multiscale cancer modeling, *in silico* medicine, clinical adaptation, decision-support system, Nephroblastoma Oncosimulator

## Abstract

**Introduction:**

Nephroblastoma or Wilms’ tumor is the most prevalent type of renal tumor in pediatric oncology. Although the overall survival rate for this condition is excellent today (∼90%), there have been no significant improvements over the past two decades. In silico models aim to simulate tumor progression and treatment responses over time; they hold immense potential for enhancing the predictive accuracy and optimizing treatment protocols as they are inspired by the digital twin paradigm.

**Methods:**

The present study uses T2-weighted magnetic resonance images, chemotherapy treatment plans, and post-surgical histological profiles from three patients enrolled in the SIOP 2001/GPOH clinical trial, where each patient represents a distinct clinically assessed risk group. We investigated the clinical adaptation of the Nephroblastoma Oncosimulator to the datasets from these patients with the goal of deriving appropriate value distributions of the model input parameters that enable accurate prediction of tumor volume reduction in response to preoperative chemotherapy.

**Results:**

Our primary focus was on the total cell kill ratio as a parameter reflecting treatment effectiveness. We derived the distribution of this parameter for one patient from each risk group: low (*Mdn* = 0.875, *IQR* [0.750, 0.875], *n* = 178), intermediate (*Mdn* = 0.875, *IQR* [0.750, 0.875], *n* = 175), and high (*Mdn* = 0.485, *IQR* [0.438, 0.532], *n* = 103). Statistically significant differences were observed between the high-risk group and both the low- and intermediate-risk groups (*p* < 0.001).

**Discussion:**

The present work establishes a foundation for further studies using available retrospective datasets and additional patients per risk group. These efforts are expected to help validate the findings, advance model development, and extend this mechanistic multiscale discretized cancer model. However, clinical validation is ultimately required to assess the potential uses of the model in clinical decision-support systems.

## 1 Introduction

Nephroblastoma or Wilms’ tumor (WT) is known to be the most common type of renal tumor in childhood and adolescence (Wilms, 1899). Approximately nine out of 10 malignant tumors in the kidneys are known to be WTs (Nakata et al., 2020). It is a rare type of pediatric cancer that has an incidence of approximately 1 in 10,000 children under the age of 15 years in Europe and North America. The median age of onset of WT is approximately 3.5 years; it occurs slightly more frequently in female patients, with a male-to-female incidence ratio of 0.9 (Steliarova-Foucher et al., 2017). Unilateral tumor cases are far more common (∼95%) than bilateral cases (∼5%) (Graf et al., 2006); in contrast, bilateral tumors are more often associated with hereditary predisposition syndromes (Dome et al., 2015; Graf et al., 2022).

WTs have been investigated and treated in prospective, randomized, and multicentric clinical studies and trials for over 50 years. These efforts are mainly attributable to the Children’s Oncology Group (COG) in North America and the International Society of Pediatric Oncology (SIOP) in Europe. While the COG favors primary surgery before chemotherapy (Geller et al., 2023), SIOP recommends preoperative chemotherapy based on medical imaging studies that assess tumor localization and metastatic status to determine the disease stage (Vujanić et al., 2018). Preoperative chemotherapy aims to reduce the tumor volume and minimize the risk of intraoperative tumor rupture. One of the key advantages of this approach is the ability to assess treatment responses before surgery; this enables stratifying the risk-adapted postoperative therapy. Patients who respond well to preoperative chemotherapy may require less intensive treatment, whereas the treatment can be intensified early for those who show poor treatment responses. One of the main drawbacks of preoperative chemotherapy in this condition is the risk of initially mistreating non-WT cases. However, the likelihood of incorrectly treating a benign tumor is below 1% (Graf et al., 2000). After surgical removal, the tumors undergo histopathological examination for determination of the histological subtype, which is based on the proportion of vital (blastema, epithelium, stroma) and non-vital (necrosis, regressive) tissue components (Vujanić et al., 2018). Both histology and tumor stage are considered when classifying the risk group, i.e., malignancy, according to current treatment protocols. This classification is crucial for guiding postoperative treatment and prognosis.

The overall survival rate for WT is excellent and exceeds 90%. However, the survival outcomes may vary by risk group, which is determined by the histology and disease stage (Spreafico et al., 2021). The 10% mortality rate among children who still die from WT primarily includes those with diffuse anaplasia and high local or overall stage after a first relapse; this trend has not changed substantially over the last 20 years. Hence, further research is needed to improve patient survival rates and increase their life expectancy as well as quality of life. If the quantitative effectiveness of treatment can be accurately predicted at the time of diagnosis, the most appropriate treatment with minimal complications can be selected as early as possible (Graf et al., 2009). Ideally, this prediction would focus on not only the chemotherapeutic responses but also the possible late effects of therapy, such as impacts on renal function and cardiotoxicity (Wright et al., 2009; Irtan et al., 2016; Weil et al., 2023).

*In silico* oncology is one approach toward this goal, where the evolution of tumor volume and its responses to preoperative chemotherapy or radiation are modeled. This approach aims to estimate treatment effectiveness and potential risks before treatment administration. The Nephroblastoma Oncosimulator (Stamatakos et al., 2007) is a computational tool that evolved from several European research projects, including ACGT (Graf et al., 2009; Stamatakos et al., 2011; Stamatakos et al., 2014; Georgiadi et al., 2012; Bucur et al., 2016), p-medicine (Georgiadi et al., 2012; Stamatakos et al., 2014; Bucur et al., 2016), ContraCancrum (Georgiadi et al., 2012; Stamatakos et al., 2014), and CHIC (Bucur et al., 2016; Kolokotroni et al., 2024). This tool is a top–down, mechanistic, and multiscale discretized cancer model designed to simulate the dynamic evolution of tumor volume in response to treatments like chemotherapy at the cellular and tissue scales. By integrating patient-specific imaging and treatment data, the model creates a digital twin of the tumor to predict its evolution over time (Stamatakos et al., 2007, 2011, 2014; Graf et al., 2009; Georgiadi et al., 2012).

In the present study, we investigated the clinical adaptation of real patient data using the Nephroblastoma Oncosimulator. As ground-truth examples, we analyzed three distinct histological profiles of WTs that each corresponded to a clinically assessed risk group. The adaptation focused on the tumor volume responses to preoperative chemotherapy, and the primary goal was to determine whether distinct and appropriate joint distributions of the input parameter values could be established for all three risk groups. We focused on the total cell kill ratio ( ${CKR}_{Total}$ ) parameter that reflects the effectiveness of preoperative chemotherapy. First, we explored the value ranges of the parameter using an optimization algorithm and evaluated the model’s ability to predict actual tumor volume reduction by quantifying the relative deviation between the predicted and observed values. Second, we incorporated histological profiles to identify representative simulation iterations; this allowed us to assess the impacts on refining suitable parameter distributions as different tumor components respond differently to chemotherapy.

Previous studies have successfully demonstrated the clinical adaptation of the model to real patient data as proof of principle (Stamatakos et al., 2011; Stamatakos et al., 2014; Georgiadi et al., 2012; Kolokotroni et al., 2024). However, the present work is a pilot study aimed at evaluating the model’s performance in relation to different risk groups and their respective histological profiles. Our evaluations are based on a prior sensitivity analysis and an error metric that serve as a method for uncertainty quantification in the simulation results. The feasibility of model adaptability is crucial for thorough clinical validation using more comprehensive datasets encompassing additional examples for all three risk groups; this is also a key factor for addressing scalability. Ultimately, such decision-support systems are expected to become valuable tools for physicians in clinical oncology to predict chemotherapeutic outcomes before treatment (Pawloski et al., 2019; Nafees et al., 2023). Such systems also require implementation through appropriate validated workflows (Bucur et al., 2016) and the necessary infrastructure (Christodoulou et al., 2016).

## 2 Materials and methods

### 2.1 Patient dataset

The patient dataset used in this work was derived from a retrospective database hosted by the Department of Pediatric Oncology and Hematology, Saarland University, Germany; this dataset was a result of the SIOP 2001/GPOH clinical trial (ClinicalTrials.gov: NCT00047138). Ethical approval for this study was obtained from the Independent Ethics Committee “Ärztekammer des Saarlandes” under the following reference numbers: 136/01 (20 September 2002; 16 September 2010; 31 March 2011), 104/10 (20 July 2010), and 248/13 (13 January 2014; 1 July 2015). The trial was conducted as a registry study from 2011 to 2022, followed by the UMBRELLA SIOP-RTSG 2016. The clinical, histological, and imaging data from one representative patient per clinically assessed risk group were retrieved from the database (Table 1): low risk (P1), intermediate risk (P2), and high risk (P3). These risk groups were determined on the basis of tumor histology after surgery and disease staging, as outlined in the SIOP protocol (SIOP 2001/GPOH, 2010).

**TABLE 1:** Wilms’ tumor (WT) characteristics of the patients as retrieved from the database along with the magnetic resonance imaging (MRI) scans and surgery dates (expressed as dd/mm/yy). The risk groups and histological subtypes were assessed after surgery according to the protocol (SIOP 2001/GPOH, 2010). ACT = actinomycin, VCR = vincristine, DOX = doxorubicin.

Patient ID	P1	P2	P3
MRI date (pre-chemo)	07/09/11	22/08/16	04/02/13
Tumor volume (pre-chemo) [cm^3^ = mL]	143.99	536.73	306.99
Start of chemotherapy	13/09/11	01/09/16	06/02/13
Treatment plan	13/09/11 (ACT, VCR, DOX) 20/09/11 (VCR) 29/09/11 (ACT, VCR) 06/10/11 (VCR) 13/10/11 (ACT, VCR, DOX) 20/10/11 (VCR)	01/09/16 (ACT, VCR) 08/09/16 (VCR) 15/09/16 (ACT, VCR) 22/09/16 (VCR)	06/02/13 (ACT, VCR) 13/02/13 (VCR) 22/02/13 (ACT, VCR) 01/03/13 (VCR)
MRI date (post-chemo)	26/10/11	23/09/16	08/03/13
Tumor volume (post-chemo) [cm^3^ = mL]	10.62	89.49	126.29
Tumor volume reduction percentage (post-chemo) [%]	92.6	83.3	58.9
Surgery date	02/11/11	27/09/16	19/03/13
Necrosis/regressive changes (macroscopic) [%]	98	70	5
Vital blastema [%]	0	20	70
Vital epithelium [%]	100	0	5
Vital stroma [%]	0	80	25
Histological subtype	Regressive	Regressive	Blastemal
Risk group	Low risk	Intermediate risk	High risk

All the tumors analyzed in this study were unilateral cases in which the subjects had received preoperative chemotherapy according to the referenced SIOP protocol. Table 1 shows the drug administration plan and a summary of further characteristics. Specifically, P2 and P3 were administered four-week treatment plans including vincristine (VCR, weekly) and actinomycin (ACT, biweekly). In contrast, P1 was administered a 6-week treatment plan including VCR, ACT, and doxorubicin (DOX), which is specific to metastatic diseases. Magnetic resonance imaging (MRI) scans were obtained in DICOM format before and after preoperative chemotherapy, and the tumor volumes were calculated using the ellipsoid formula, as outlined in the SIOP protocol. All three patients responded well to the administered preoperative chemotherapy, as indicated by tumor volume reductions of more than 50%. However, P3 was notable owing to the unfavorable tumor histology characterized by predominantly treatment-resistant vital blastema (Table 1); this results in worse prognosis, as indicated by the high-risk group classification. To better understand this relationship, it is essential to consider that proliferative cells are more susceptible to chemotherapy than differentiated cells as they divide actively and are more likely to be affected by cytotoxic agents. Blastema is highly proliferative and contrasts with the epithelial and stromal components that contain higher fractions of differentiated cells. This explains why chemotherapy-resistant blastema persistence after treatment is a critical risk factor for poor prognosis.

The postoperative histological tumor profiles of the patients provide insights into the initial cellular compositions of the tumors at the time of diagnosis. Post-chemotherapy necrosis and regressive changes likely arise from the initial blastema that was effectively targeted by chemotherapy. Together with the remaining vital blastema, this allows estimation of the tumor’s minimum initial fraction of proliferative cells (IFPC) prior to the start of chemotherapy. This minimum value reflects the possibility that other proliferative tumor cells may have also responded to chemotherapy and are thus not accounted for in this estimation. The IFPC remains an approximation as the tumor’s initial histological composition before treatment is unknown and histopathological assessments are typically performed after surgical resection. Similarly, the initial presence of necrotic tissues cannot be determined with certainty in retrospect. These necrotic tissues may have resulted from insufficient vascularization, oxygen supply, and nutrient availability rather than therapy. Cysts and hemorrhages are also excluded from this estimation. Despite these limitations, this approach provides a reasonable approximation of the tumor’s estimated IFPC, given by Equation 1.

$$\text{IFP}C=\frac{\left(V_{post-chemo}\times f_{regressive\;changes}\right)+\left(V_{post-chemo}\times f_{vital\;tissue}\times f_{vital\;blastema}\right)\;.}{V_{pre-chemo}}$$ (1)

This results in the following minimum fractions for the three patients: P1 (IFPC) = 8.2%, P2 (IFPC) = 10.2%, and P3 (IFPC) = 32.8%. These lower bounds are defined as the histology criterion.

### 2.2 Image data preprocessing

The original T2-weighted MRI scans of the patients were preprocessed as inputs to the Nephroblastoma Oncosimulator. This preprocessing step is necessary to address the heterogeneous scanner settings (Table 2) and to convert the scans into the input format required by the model. Although the SIOP protocol specifies the imaging dates and modalities, the device-specific settings may still vary. The MRI scans were converted from DICOM to NRRD format using 3D Slicer (Version 4.10.2) (Fedorov et al., 2012). The tumor regions for each patient were segmented manually and annotated on the image slices using MITK Workbench (Version 2018.04) (Wolf et al., 2005). These annotations were then validated and verified by a pediatric oncology expert. Based on the annotations, 3D Slicer was used to assign binary labels (0 = non-tumor tissue, 1 = tumor tissue) to each of the voxels of the tumor’s 3D discretization. As a result, the WT in each patient was represented as a 3D matrix of discrete geometrical cells, where each geometrical cell consists of multiple biological cells grouped into equivalence classes in the model. Cells within the same equivalence class are assumed to undergo identical cytokinetic transitions within the cell cycle (Stamatakos et al., 2014). The original voxel spacing (Table 2) was standardized to 1 mm along all image axes using the integrated resampling method with nearest neighbor interpolation available in the software. The final 3D tumor representations were saved in the common MetaIO standard (FAIRsharing, 2015).

**TABLE 2:** Original voxel spacing in MRI scans before resampling for standardization during preprocessing. MRI = magnetic resonance imaging.

Patient ID	P1		P2		P3	
Risk group	Low risk		Intermediate risk		High risk	
Spacing of voxels along the image axes	Pre-chemo	Post-chemo	Pre-chemo	Post-chemo	Pre-chemo	Post-chemo
x-axis [mm]	0.9375	0.9375	1.125	1.031	0.7813	0.6875
y-axis [mm]	0.9375	0.9375	1.125	1.031	0.7813	0.6875
z-axis [mm]	5.5	5.5	6.5	6.5	3.2999	4.4

### 2.3 Nephroblastoma Oncosimulator

Simulations conducted using the Nephroblastoma Oncosimulator provide *in silico* predictions of tumor evolution, including both growth and regression as well as treatment responses over time, where the model is based on a 3D representation of a patient’s tumor. The input parameters regulate the cell population kinetics at both cellular and tissue scales. The model incorporates the cell cycle and accounts for varying proportions of stem cells, progenitor cells, differentiated cells, and dead cells (apoptotic and necrotic). The complex interactions among the proliferative, apoptotic, and necrotic processes are simulated in a parameterized manner; by considering these factors, the model captures the dynamics of tumor volume development, including treatment-induced shrinkage.

**FIGURE 1:**
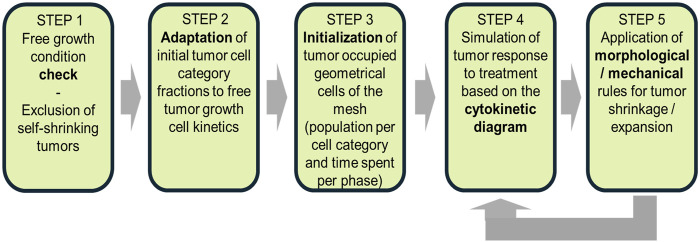
Flowchart of the Oncosimulator procedures for a macroscopically homogeneous nephroblastoma of arbitrary shape. The diagram outlines key steps from tumor initialization to treatment response simulation, including the morphological and mechanical adaptations.

Figure 1 presents a flowchart illustrating the model’s simulation algorithm. The key steps of the process are outlined below. A comprehensive description of the generic simulation algorithm was published previously by Stamatakos et al. (2025), but its distinction from the current study is that the radiotherapy treatment described therein is replaced by the chemotherapy regimen.

• Tumor definition (STEP 0): The mesh initialization phase involves defining the occupied and non-occupied geometrical cells based on available patient-specific imaging data.
• Free growth condition check (STEP 1): A condition is applied to assess whether the given combination of input parameters leads to tumor growth or spontaneous regression. Tumors that fail to exhibit sustained growth are then excluded from the model.
• Adaptation of tumor cell category fractions (STEP 2): The initial distribution of tumor cell categories is adjusted to align with the kinetics of untreated tumor growths.
• Initialization of tumor-occupied mesh cells (STEP 3): The geometrical cells occupied by the tumor tissues are initialized by incorporating the population per cell category and phase duration.
• Tumor response simulation (STEP 4): This simulation accounts for the tumor’s response to treatment based on the cytokinetic diagram. This step involves the first complete scan of the mesh.
• Morphological and mechanical adaptations (STEP 5): The morphological and mechanical rules governing tumor growth, shrinkage, and structural changes are applied during a second mesh scan.

This structured framework underpins the *in silico* modeling of tumor evolution by allowing dynamic adjustments based on patient-specific clinical data.

#### 2.3.1 Model parameters

The Nephroblastoma Oncosimulator produces a spatial voxel-based representation of the tumor and its microenvironment based on MRI scans before and after chemotherapy as well as a set of input parameters defining the treatment characteristics and cell cycle dynamics. These elements collectively influence tumor growth, therapy response, and the overall simulation outcome. The treatment-specific input parameters are derived from patient-specific clinical data, e.g., timing of chemotherapy drug administration and total treatment duration. These time points are aligned with the MRI scan dates before and after chemotherapy (Georgiadi et al., 2012) (Table 3). Another key set of parameters is used to map the cell cycle dynamics (Table 4) (Stamatakos et al., 2011; Georgiadi et al., 2012). For example, $P_{sym}$ and $P_{sleep}$ regulate tumor cell division and quiescence to influence tumor aggressiveness by affecting the proportions of differentiated, proliferative, and dormant cells. Here, $T_{d}$ represents the tumor doubling time.

**TABLE 3:** Input parameters to simulations with the Nephroblastoma Oncosimulator and their values derived from clinical data. As these are patient-specific parameter values, they remain unchanged for all VPs during optimization for clinical adaptation. MRI = magnetic resonance imaging, VP = virtual patient, VCR = vincristine, ACT = actinomycin.

Input parameter	Description	P1	P2	P3
VCR-ADMIN-A	Time point of first VCR administration (days after MRI pre-chemo)	4	4	4
VCR-ADMIN-B	Time point of second VCR administration (days after MRI pre-chemo)	12	11	11
VCR-ADMIN-C	Time point of third VCR administration (days after MRI pre-chemo)	19	18	20
VCR-ADMIN-D	Time point of fourth VCR administration (days after MRI pre-chemo)	26	25	27
ACT-ADMIN-A	Time point of first ACT administration (days after MRI pre-chemo)	4	4	4
ACT-ADMIN-B	Time point of second ACT administration (days after MRI pre-chemo)	19	18	20
DT-post-treat	Duration in days between last drug administration (ACT-ADMIN-B) and time point of simulation completion (MRI post-chemo)	6	1	7

**TABLE 4:** Reference values for the input parameters of the simulation with the Nephroblastoma Oncosimulator, which serve as foundation for clinical adaptation for all three patients. d = days, h = hours, STEM = stem cells, LIMP = cell with limited mitotic potential, CKR = cell kill ratio.

Input parameter	Description	References value	Unit	References
T_d_	Doubling time of the tumor (size and cell population)	29	d	Stamatakos et al. (2011)
T_c_	Cell cycle duration	23	h	Revazova and Petrova (1981)
T_G0_	Time required for dormant cells to die through necrosis	96	h	Måseide and Rofstad (2000)
T_N_	Time required for complete necrosis and removal of the products from the tumor	20	h	Düchting et al. (1992); Wein et al. (2000); Anderson et al. (2006)
T_A_	Time required for complete apoptosis and removal of the products from the tumor	6	h	Dewey et al. (1995); Ribba et al. (2006)
R_A_	Percentage of undifferentiated cells that die by apoptosis per hour (STEM and LIMP)	0.001	1/h	Dewey et al. (1995); Ribba et al. (2006)
R_ADiff_	Percentage of differentiated cells that die by apoptosis per hour	0.003	1/h	Dewey et al. (1995); Ribba et al. (2006)
R_NDiff_	Percentage of differentiated cells that die by necrosis per hour	0.001	1/h	Düchting et al. (1992); Wein et al. (2000); Anderson et al. (2006)
P_G0toG1_	Percentage of undifferentiated cells leaving the resting G_0_ phase to re-enter the cell cycle (STEM and LIMP)	0.01	1/h	
N_LIMP_	Maximum number of mitoses that a LIMP cell can undergo before terminal differentiation	3	-	
P_sym_	Percentage of stem cells that divided symmetrically	0.45	1/h	
P_sleep_	Percentage of cells entering the resting G_0_ phase after mitosis (STEM and LIMP)	0.28	1/h	
CKR_VCR_	Fraction of tumor cells affected by the administered vincristine drug dose	0.3		Dahl et al. (1976); Groninger et al. (2002)
CKR_ACT_	Fraction of tumor cells affected by the administered actinomycin drug dose	0.2		Sawada et al. (2005); Veal et al. (2005)
CKR_Total_	Overall fraction of tumor cells affected by the administered chemotherapy	0.5		

The chemotherapeutic response parameters quantify the effectiveness of treatment, where ${CKR}_{VCR}$ and ${CKR}_{ACT}$ represent the drug-specific effects of VCR and ACT, respectively. ${CKR}_{Total}$ indicates the overall impact of a four-week chemotherapeutic treatment plan with ACT and VCR, according to the SIOP protocol; it indicates the fraction of tumor cells eradicated by treatment and is defined as the sum of its drug-specific components (Table 4; Equation 2).

$$\begin{array}{c} \;{CKR}_{Total}={CKR}_{ACT}+{CKR}_{VCR}\;\\ \;{CKR}_{ACT}=\left(3/5\right)*{CKR}_{Total}\\ \;{CKR}_{VCR}=\left(2/5\right)*{CKR}_{Total} \end{array}$$ (2)

A higher ${CKR}_{Total}$ value leads to increased apoptosis, reducing the proliferative and dormant cell populations while increasing the fraction of differentiated and dead cells. The output parameters of the model (Table 5) include the predicted tumor volume reduction percentage as well as the initial and final tumor compositions categorized into five different cell populations.

**TABLE 5:** Output parameters of the simulation with the Nephroblastoma Oncosimulator, including the computed initial percentages of different cell types at the start of simulation and final percentages at the end of simulation.

Output parameter	Description
PROLIF_Initial_	Initial percentage of proliferative cells
DORMANT_Initial_	Initial percentage of dormant cells
DIFF_Initial_	Initial percentage of differentiated cells
DEAD_Initial_	Initial percentage of dead cells
PROLIF_Final_	Final percentage of proliferative cells
DORMANT_Final_	Final percentage of dormant cells
DIFF_Final_	Final percentage of differentiated cells
DEAD_Final_	Final percentage of dead cells
DV	Final percentage of tumor reduction

Sensitivity analyses have been previously used to examine the impacts of the input parameters on the simulation outcomes (Stamatakos et al., 2011; Stamatakos et al., 2014; Georgiadi et al., 2012). These analyses showed that ${CKR}_{Total}$ has the strongest influence on tumor volume response to chemotherapy. More details on the sensitivity analyses as well as performance-related aspects, such as application profiling, and code optimization for high-performance simulations (Panagiotidou et al., 2022) are beyond the scope of this study as the present focus is on clinical, adaptation.

#### 2.3.2 Clinical adaptation

The goal of clinical adaptation is to determine a joint distribution of the input parameter values that maximizes the accuracy of the predicted tumor volume reduction, i.e., the real percentage of tumor volume reduction observed clinically for a patient. This process involves performing a predefined number (*N*) of simulations, which are referred to as virtual patients (VPs), for each patient in each risk group. As a proof of principle, we explored two different sample sizes of VPs per patient, i.e., *N* = 20 and *N* = 200. To evaluate the accuracies of the simulations, we used the relative deviation between the predicted and observed (ground-truth) tumor volume reductions. This deviation is referred to as the clinical adaptation error. A maximum deviation of 5% was set as the threshold for the adaptation criterion based on the recommendation of a pediatric oncology expert. This threshold balances tumor volume deviations with biological variability and imaging uncertainties.

For each VP, an optimization algorithm is used to determine a value for a selected adaptation parameter that satisfies the adaptation criterion while retaining fixed values for all other parameters. The algorithm follows a binary search approach, where the adaptation parameter is initially set to the median value between predefined lower and upper bounds before conducting the simulation. The algorithm terminates when the predicted tumor volume reduction meets the adaptation criterion. If the predicted tumor volume reduction exceeds the ground-truth value, the current adaptation parameter value is set as the new upper bound; conversely, if the predicted tumor volume reduction is less than the ground-truth value, the current value becomes the new lower bound. The algorithm iterates until the adaptation criterion is met or the bounds converge. In the latter case, no suitable value is found for the adaptation parameter.

In each iteration, the algorithm assigns an updated median value to the adaptation parameter that is quantified by the clinical adaptation error of the simulation. The distribution of the adaptation parameter values for each VP is then determined by analyzing the frequency of values assigned to the input parameters. The final iteration of the algorithm for each VP typically provides an optimized value for the adaptation parameter corresponding to a simulation outcome satisfying the adaptation criterion. If no suitable value is found, it is considered that the input parameter values did not allow the adaptation criterion to be met. The results from all iterations of the optimization algorithm are evaluated for all VPs of each patient. The distribution of the adaptation parameter values is determined by analyzing the frequency of assigning values in all iterations. Each simulation execution is performed with a specific adaptation parameter value that is characterized by its corresponding clinical adaptation error. Both the adaptation criterion and histology criterion (see Section 2.1) can be applied to further refine the permissible distribution of the adaptation parameter values by excluding simulations that do not satisfy these criteria.

In the present study, ${CKR}_{Total}$ was chosen as the adaptation parameter given its substantial influence on the model outcome. During the first iteration of the optimization, the lower bound was set to 0 while the upper bound was set to 1. The treatment-specific input parameter values were retrieved from the clinical data for each patient in each of the risk groups (Table 1) and were fixed for all VPs of a given patient (Table 3). P1 was simulated in the same manner as P2 and P3 following a 4-week treatment regimen starting on 29 September 2011 by excluding the administration of DOX.

Input parameters representing the cell cycle dynamics (Table 4) were assigned individually for each VP. These values remained fixed over all iterations of the optimization algorithm for each VP. Since no experimental data were available for the clinical patient cohort for these parameters, we used reference values from literature (Stamatakos et al., 2011; Georgiadi et al., 2012). These reference values served as the central values for uniform probability distributions, from which the parameter values were drawn. A maximum deviation of 50% from the reference value was allowed to ensure sufficient exploration of the parameter space; this method enabled systematic variations of parameter values across multiple VPs, increasing the robustness of the simulation to account for biological diversity (Stamatakos et al., 2011; Stamatakos et al., 2014; Georgiadi et al., 2012; Panagiotidou et al., 2022).

The model simulations were implemented in C++ (version C++14). All simulations were conducted on a Linux server with two Intel® Xeon® CPU E5-2658 A v3 @2.20 GHz processors. Each processor contained twelve physical cores running two threads each, resulting in a total of 48 CPUs. This setup enabled parallel executions and minimized the runtime of the computationally intensive tasks.

#### 2.3.3 Statistical analysis

Statistical analysis was conducted using IBM SPSS Statistics (Version 28.0.1.1). Two-sided *p*-values <0.05 were considered to be statistically significant. Since the assumption of normality was not met, non-parametric tests were applied. The Mann–Whitney U test was used to compare distributions between two samples. The Kruskal–Wallis H test, a non-parametric alternative to ANOVA, was applied to comparisons involving more than two samples. The *post hoc* analyses included pairwise comparisons using Dunn’s method with Bonferroni correction of *p*-values for multiple tests. To quantify the effect size for the pairwise comparisons, Pearson’s r value was calculated using the standard test statistics z and sample size (Fritz et al., 2012). The effect sizes of |r| = 0.1, 0.3, and 0.5 were interpreted as small, medium, and large effects, respectively (Cohen, 2013).

## 3 Results

### 3.1 Image data preprocessing

Table 6 shows the tumor volumes from the SIOP 2001/GPOH database and the manually segmented 3D tumor representations. These results are from the preprocessed MRI scans before and after preoperative chemotherapy for the three patients. The database volumes are approximations based on the ellipsoid formula. In contrast, the segmentation volumes are directly computed from the 3D representations of the manually segmented and preprocessed MRI scans of tumors. The data reveal some variability between the two methods, particularly in the prechemotherapeutic tumor volumes. For example, the database reports a volume of 143.99 cm^3^ for P1, whereas manual segmentation yields a volume of 126.25 cm^3^; the post-chemotherapy volumes differ to a lesser degree as both methods provide more similar estimates.

**TABLE 6:** Overview of the tumor volume data retrieved from the retrospective database (calculated using the ellipsoid formula) and the volumes of manually segmented tumors from the preprocessed MRI scans. MRI = magnetic resonance imaging.

Patient ID	P1		P2		P3	
Risk group	Low risk		Intermediate risk		High risk	
	Database	Segmentation	Database	Segmentation	Database	Segmentation
Tumor volume (pre-chemo) [cm^3^ = mL]	143.99	126.25	536.73	526.99	306.99	235.64
Tumor volume (post-chemo) [cm^3^ = mL]	10.62	10.58	89.49	70.72	126.29	108.22
Tumor volume reduction percentage (post-chemo) [%]	92.6	91.6	83.3	86.6	58.9	54.1

### 3.2 Clinical adaptation with the Nephroblastoma Oncosimulator

**FIGURE 2:**
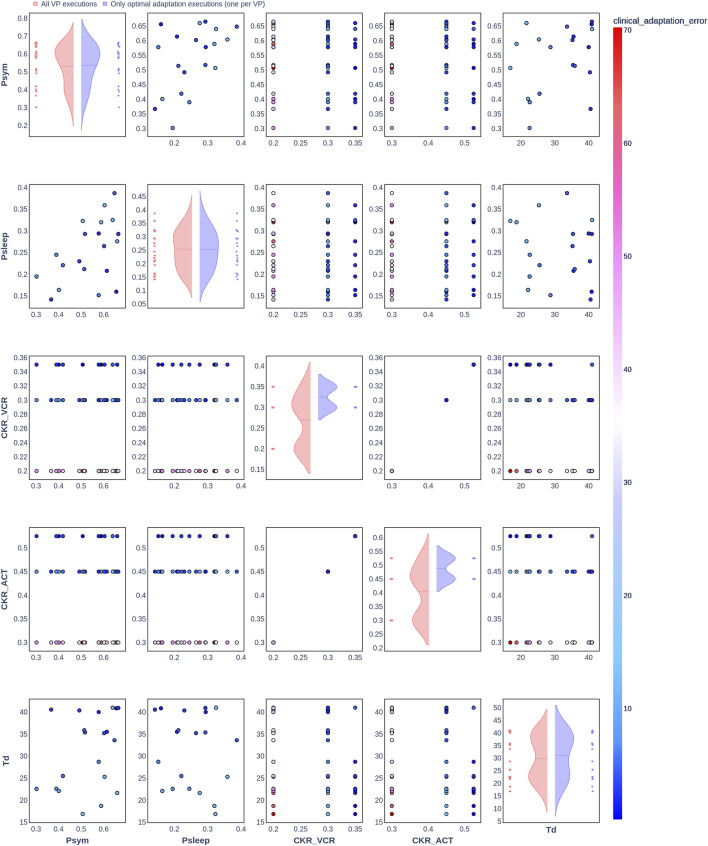
Pair plot showing the joint distribution of values assigned to the input parameters of the simulation during iteration of the clinical adaptation optimization algorithm for patient P1 (*N* = 20 VPs). The dots in the scatter plot are color-coded to represent the resulting clinical adaptation errors for the assigned values. Here, optimal adaptation refers to the final iteration of the optimization algorithm. VP = virtual patient.

**FIGURE 3:**
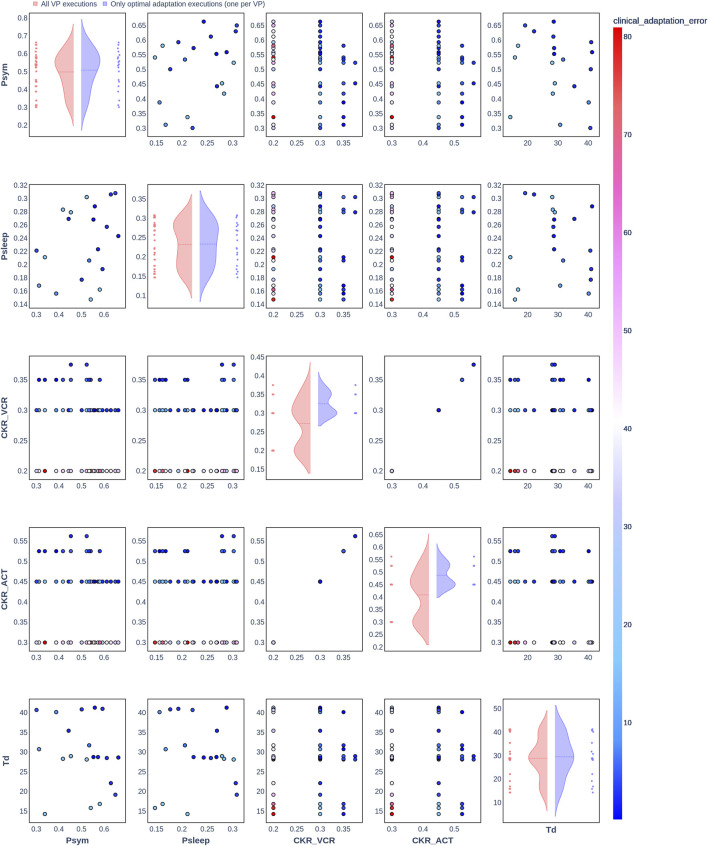
Pair plot showing the joint distribution of values assigned to the input parameters of the simulation during iteration of the clinical adaptation optimization algorithm for patient P2 (*N* = 20 VPs). The dots in the scatter plot are color-coded to represent the resulting clinical adaptation errors for the assigned values. Here, optimal adaptation refers to the final iteration of the optimization algorithm. VP = virtual patient.

**FIGURE 4:**
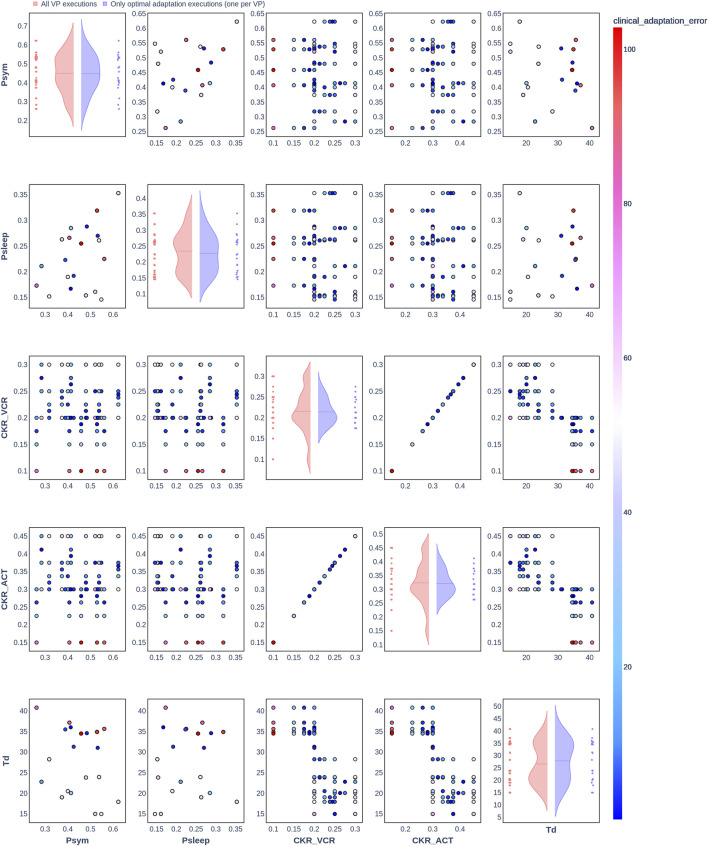
Pair plot showing the joint distribution of values assigned to the input parameters of the simulation during iteration of the clinical adaptation optimization algorithm for patient P3 (*N* = 20 VPs). The dots in the scatter plot are color-coded to represent the resulting clinical adaptation errors for the assigned values. Here, optimal adaptation refers to the final iteration of the optimization algorithm. VP = virtual patient.

**FIGURE 5:**
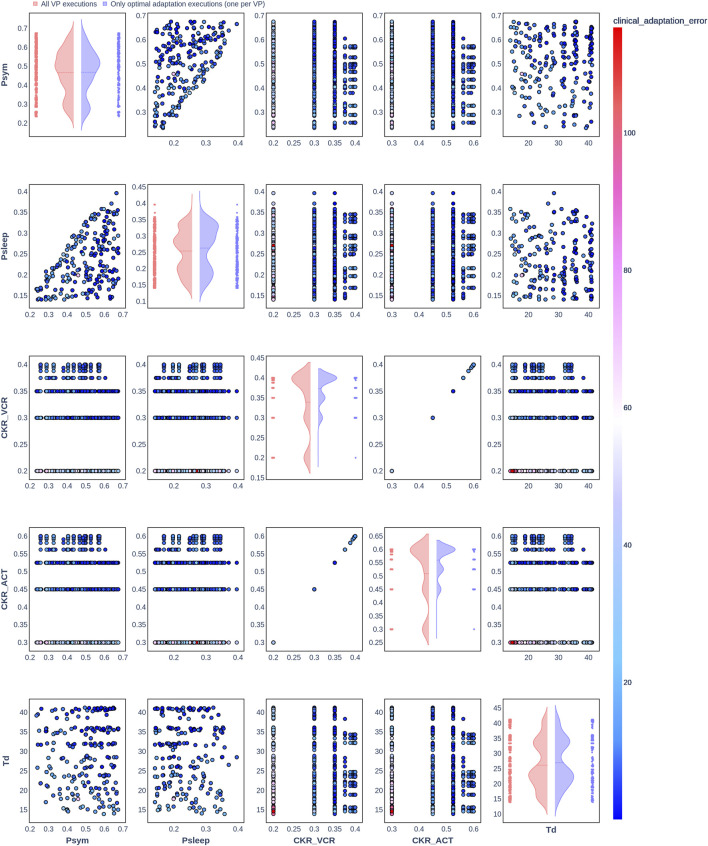
Pair plot showing the joint distribution of values assigned to the input parameters of the simulation during iteration of the clinical adaptation optimization algorithm for patient P1 (*N* = 200 VPs). The dots in the scatter plot are color-coded to represent the resulting clinical adaptation errors for the assigned values. Here, optimal adaptation refers to the final iteration of the optimization algorithm. VP = virtual patient.

**FIGURE 6:**
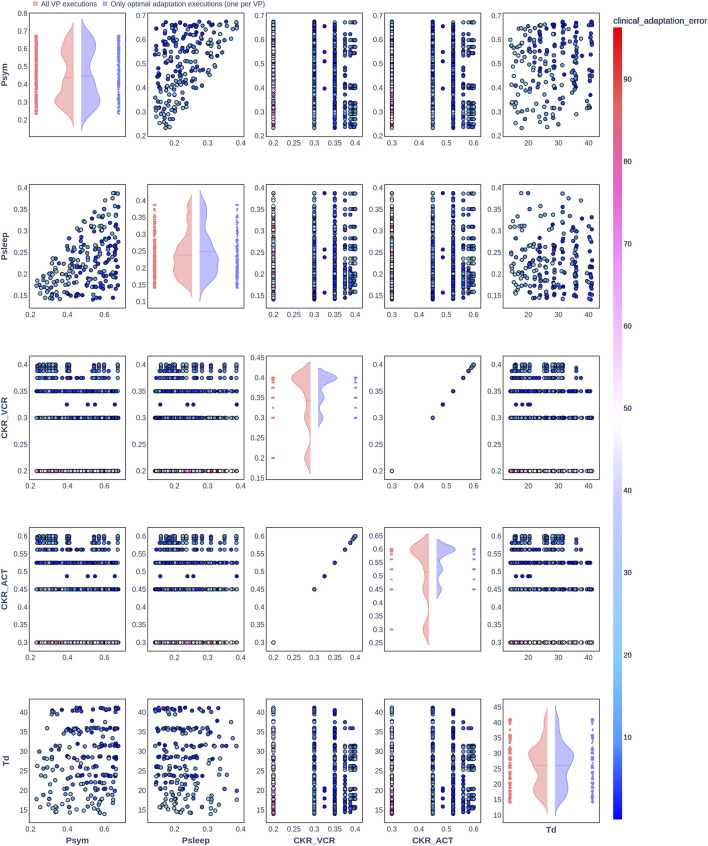
Pair plot showing the joint distribution of values assigned to the input parameters of the simulation during iteration of the clinical adaptation optimization algorithm for patient P2 (*N* = 200 VPs). The dots in the scatter plot are color-coded to represent the resulting clinical adaptation errors for the assigned values. Here, optimal adaptation refers to the final iteration of the optimization algorithm. VP = virtual patient.

**FIGURE 7:**
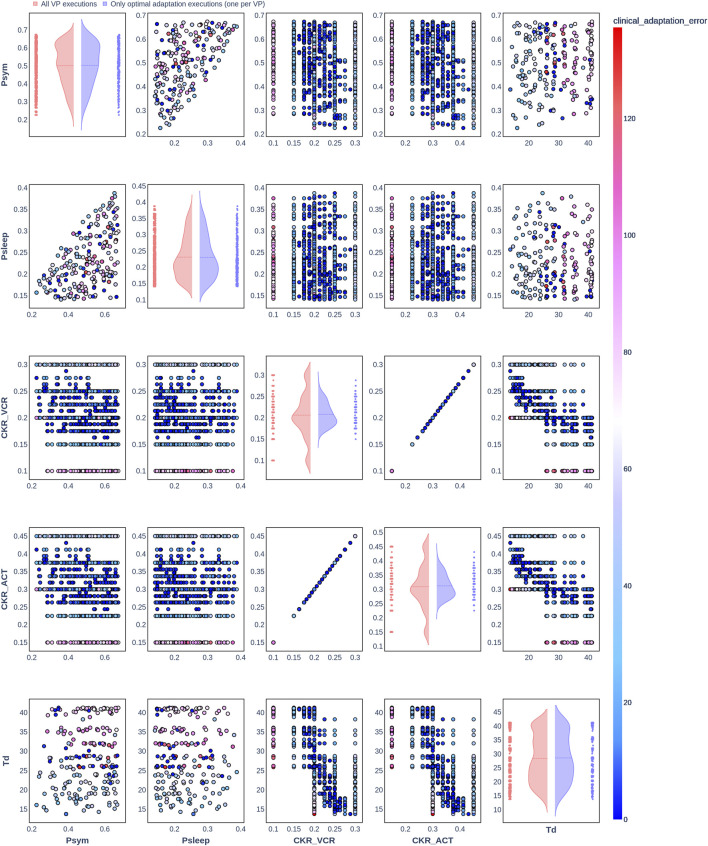
Pair plot showing the joint distribution of values assigned to the input parameters of the simulation during iteration of the clinical adaptation optimization algorithm for patient P3 (*N* = 200 VPs). The dots in the scatter plot are color-coded to represent the resulting clinical adaptation errors for the assigned values. Here, optimal adaptation refers to the final iteration of the optimization algorithm. VP = virtual patient.

The results from the optimization algorithm are visualized for each patient using pair plots consisting of scatter plots (Figures 2–7). These plots include the input parameters $P_{sym}$ , $P_{sleep}$ , ${CKR}_{VCR}$ , ${CKR}_{ACT}$ , and $T_{d}$ . Here, $P_{sym}$ , $P_{sleep}$ , and $T_{d}$ were also included in the plots to assess the degree to which the uniform distributions of these parameters were covered. The rows and columns correspond to the input parameters, and the scatter plots display the joint distributions of the corresponding parameter values. The color of the data points in the scatter plots indicates the resulting clinical adaptation error for the combination of values, ranging from blue (low error) to red (high error).

The diagonal plots depict the probability distributions of each of the input parameters during the iterations of the optimization algorithm. In these plots, the red curves represent values assigned over all iterations of the optimization algorithm for all VPs of a patient. In contrast, the blue curves display only values from the final iterations of all VPs. The number of data points in each scatter plot corresponds to the total number of optimization iterations across all VPs. The red and blue horizontal lines indicate the mean values of the distributions for each of the input parameters. The first three figures refer to *N* = 20 VPs for P1, P2, and P3 (Figures 2–4, respectively), while the last three figures refer to *N* = 200 VPs for P1, P2, and P3 (Figures 5–7, respectively). The color-coded clinical adaptation errors in the scatter plots reveal that not all joint distributions of the parameter values satisfy the adaptation criterion. As the number of VPs increases, the value ranges of the uniform distributions for the parameters $P_{sym}$ , $P_{sleep}$ , and $T_{d}$ are represented more extensively. The scatter plots for ${CKR}_{VCR}$ and ${CKR}_{ACT}$ demonstrate their dependency, as evidenced by the distribution of the data points.

Table 7 presents the distribution of explored value ranges for the parameters ${CKR}_{Total}$ , ${CKR}_{ACT}$ , and ${CKR}_{VCR}$ derived from the final iterations of the optimization algorithm, as shown in the respective diagonal plots in Figures 2–7. The distributions are categorized according to the numbers of VPs explored per patient (*N* = 20, *N* = 200). The ranges of the respective clinical adaptation errors confirmed that not all simulations in the final iteration of the optimization algorithm with *N* = 200 satisfied the adaptation criterion except for P3. Specifically, 11 out of 200 VPs (5.5%) for P1 did not meet the adaptation criterion, while 12 out of 200 VPs (6%) for P2 did not meet the criterion and had a maximum clinical adaptation error of approximately 19.5%. In contrast, the adaptation criterion was met for all three patients when *N* = 20. The median values of ${CKR}_{Total}$ and clinical adaptation error decreased with increasing risk groups. This pattern was not observed for *N* = 200, where the low- and intermediate-risk groups exhibited similar median values. The Kruskal–Wallis H test indicated that the distributions of ${CKR}_{Total}$ values differed significantly among the three patients for *N* = 20 (χ^2^ (2, *N* = 60) = 42.14, *p* < 0.001). Post hoc comparisons also indicated significant differences with a large effect size between P1 and P3 (*z* = 5.62, *p* < 0.001, |*r*| = 0.89) as well as between P2 and P3 (*z* = −5.62, *p* < 0.001, |*r*| = 0.89), while no significant difference was observed between P1 and P2. For *N* = 200, the distributions of ${CKR}_{Total}$ values also differed significantly among the three patients (χ^2^ (2, *N* = 600) = 418.3, *p* < 0.001). Post-hoc comparisons confirmed significant differences with a large effect size between P1 and P3 (*z* = 17.60, *p* < 0.001, |*r*| = 0.88) as well as between P2 and P3 (*z* = −17.82, *p* < 0.001, |*r|* = 0.89), while no significant difference was observed between P1 and P2. The Mann–Whitney U test revealed that the distributions of ${CKR}_{Total}$ values did not differ significantly between *N* = 20 and *N* = 200 for all three patients.

**TABLE 7:** Distributions of the adapted CKR parameters and corresponding clinical adaptation errors for the three patients obtained from the final iteration of the optimization algorithm for the VPs. CKR = cell kill ratio, VP = virtual patient, *Mdn* = median, *IQR* = interquartile range, *Min* = minimum, *Max* = maximum.

Patient ID	P1	P2	P3
Risk group	Low risk	Intermediate risk	High risk
*N* = 20			
${CKR}_{ACT}$	*Mdn* = 0.488 *IQR* = [0.450, 0.525] *Min* = 0.450 *Max* = 0.525	*Mdn* = 0.450 *IQR* = [0.450, 0.525] *Min* = 0.450 *Max* = 0.562	*Mdn* = 0.310 *IQR* = [0.286, 0.364] *Min* = 0.263 *Max* = 0.412
${CKR}_{VCR}$	*Mdn* = 0.325 *IQR* = [0.300, 0.350] *Min* = 0.300 *Max* = 0.350	*Mdn* = 0.300 *IQR* = [0.300, 0.350] *Min* = 0.300 *Max* = 0.375	*Mdn* = 0.207 *IQR* = [0.191, 0.243] *Min* = 0.175 *Max* = 0.275
${CKR}_{Total}$	*Mdn* = 0.813 *IQR* = [0.750, 0.875] *Min* = 0.750 *Max* = 0.875	*Mdn* = 0.750 *IQR* = [0.750, 0.875] *Min* = 0.750 *Max* = 0.937	*Mdn* = 0.516 *IQR* = [0.477, 0.606] *Min* = 0.438 *Max* = 0.687
Clinical adaptation error [%]	*Mdn* = 2.5 *IQR* = [1.4, 3.5] *Min* = 0.2 *Max* = 4.9	*Mdn* = 2.3 *IQR* = [1.0, 3.2] *Min* = 0.1 *Max* = 4.3	*Mdn* = 2.2 *IQR* = [0.95, 3.8] *Min* = 0.2 *Max* = 5.0
*N* = 200			
${CKR}_{ACT}$	*Mdn* = 0.525 *IQR* = [0.450, 0.525] *Min* = 0.450 *Max* = 0.600	*Mdn* = 0.525 *IQR* = [0.450, 0.525] *Min* = 0.450 *Max* = 0.600	*Mdn* = 0.300 *IQR* = [0.281, 0.337] *Min* = 0.225 *Max* = 0.431
${CKR}_{VCR}$	*Mdn* = 0.350 *IQR* = [0.300, 0.350] *Min* = 0.300 *Max* = 0.400	*Mdn* = 0.350 *IQR* = [0.300, 0.350] *Min* = 0.300 *Max* = 0.400	*Mdn* = 0.200 *IQR* = [0.188, 0.225] *Min* = 0.150 *Max* = 0.288
${CKR}_{Total}$	*Mdn* = 0.875 *IQR* = [0.750, 0.875] *Min* = 0.750 *Max* = 1.000	*Mdn* = 0.875 *IQR* = [0.750, 0.875] *Min* = 0.750 *Max* = 1.000	*Mdn* = 0.500 *IQR* = [0.469, 0.562] *Min* = 0.375 *Max* = 0.719
Clinical adaptation error [%]	*Mdn* = 2.1 *IQR* = [1.0, 3.6] *Min* = 0.03 *Max* = 15.4	*Mdn* = 2.8 *IQR* = [1.3, 4.1] *Min* = 0.01 *Max* = 19.5	*Mdn* = 2.1 *IQR* = [0.8, 3.4] *Min* = 0.00 *Max* = 5.0

Table 8a presents the distribution of the explored values ranges for ${CKR}_{Total}$ based on the subset of final iterations for which the adaptation criterion was met. This resulted in reduced numbers (*n*) of the 200 eligible VPs per patient used to define the distribution of ${CKR}_{Total}$ values. Since the number of eligible VPs remained unchanged for *N* = 20, the statistical analysis was not repeated for this case as the previous tests remained valid. Regarding the eligible VPs from *N* = 200, the high-risk group exhibited the lowest median ${CKR}_{Total}$ value, while the low- and intermediate-risk groups displayed similar median values. The distributions of the ${CKR}_{Total}$ values differed significantly among the three patients (χ^2^ (2, *N* = 577) = 412.3, *p* < 0.001). Post hoc comparisons confirmed significant differences with a large effect size between P1 and P3 (*z* = 17.42, *p* < 0.001, |*r*| = 0.88) as well as between P2 and P3 (*z* = −17.57, *p* < 0.001, |*r*| = 0.89), but no significant difference was observed between P1 and P2. The Mann–Whitney U test revealed that the distributions of the ${CKR}_{Total}$ values did not differ significantly between the two subsets of eligible VPs derived from *N* = 20 and *N* = 200 for all three patients. However, the distributions of the clinical adaptation error rates differed significantly between the three patients for the eligible VPs from *N* = 200 (χ^2^ (2, *N* = 577) = 8.6, *p* = 0.014). Post hoc comparisons revealed significant differences with a small effect size between P1 and P2 (*z* = −2.52, *p* = 0.036, |*r*| = 0.13) as well as between P2 and P3 (*z* = −2.56, *p* = 0.031, |*r*| = 0.15), whereas no significant difference was observed between P1 and P3. The median clinical adaptation error was highest at 2.7% for P2 .

**TABLE 8:** Distributions of the adapted CKR parameters and corresponding clinical adaptation errors obtained from the final iteration of the optimization algorithm for the VPs satisfying (a) the adaptation criterion and (b) both adaptation and histology criteria. CKR = cell kill ratio, VP = virtual patient, *Mdn* = median, *IQR* = interquartile range, *Min* = minimum, *Max* = maximum, *n* = number of VPs satisfying the criteria.

Patient ID	P1		P2		P3	
Risk group	Low risk		Intermediate risk		High risk	
	*N* = 20	*N* = 200	*N* = 20	*N* = 200	*N* = 20	*N* = 200
${CKR}_{Total}$ (a)						
*Mdn* *IQR* *Min* *Max* *n*	0.813 [0.750, 0.875] 0.750 0.875 20	0.875 [0.750, 0.875] 0.750 0.992 189	0.750 [0.750, 0.875] 0.750 0.937 20	0.875 [0.750, 0.875] 0.750 0.998 188	0.516 [0.477, 0.606] 0.438 0.687 20	0.500 [0.469, 0.562] 0.375 0.719 200
Clinical adaptation error [%] (a)						
*Mdn* *IQR* *Min* *Max* *n*	2.5 [1.4, 3.5] 0.2 4.9 20	2.0 [1.0, 3.3] 0.03 5.0 189	2.3 [1.0, 3.2] 0.1 4.3 20	2.7 [1.3, 3.9] 0.01 5.0 188	2.2 [0.9, 3.8] 0.2 5.0 20	2.1 [0.8, 3.4] 0.00 5.0 200
${CKR}_{Total}$ (b)						
*Mdn* *IQR* *Min* *Max* *n*	0.875 [0.750, 0.875] 0.750 0.875 19	0.875 [0.750, 0.875] 0.750 0.992 178	0.750 [0.750, 0.875] 0.750 0.937 18	0.875 [0.750, 0.875] 0.750 0.998 175	0.532 [0.500, 0.625] 0.438 0.625 7	0.485 [0.438, 0.532] 0.375 0.657 103
Clinical adaptation error [%]						
*Mdn* *IQR* *Min* *Max* *n*	2.4 [1.2, 3.0] 0.21 4.9 19	2.1 [1.0, 3.2] 0.03 5.0 178	2.1 [0.9, 3.0] 0.1 4.2 18	2.7 [1.2, 4.0] 0.01 5.0 175	2.3 [1.0, 3.4] 0.2 4.0 7	2.2 [0.9, 3.5] 0.00 5.0 103

Table 8b considers only the VPs for which both the adaptation criterion and histology criterion (see Section 2.1) were met. The number of eligible VPs (*n*) per patient decreased further, particularly for P3, where 13 out of 20 VPs (65%) and 97 out of 200 VPs (48.5%) failed to meet both criteria. The high-risk group also exhibited the lowest median ${CKR}_{Total}$ value, while the low- and intermediate-risk group displayed similar median values. For the eligible VPs from *N* = 20, the distributions ${CKR}_{Total}$ values differed significantly among the three patients (χ^2^ (2, *N* = 44) = 19.8, *p* < 0.001). Post hoc comparisons confirmed significant differences with a large effect size between P1 and P3 (*z* = 4.19, *p* < 0.001, |*r*| = 0.82) as well as between P2 and P3 (*z* = −4.08, *p* < 0.001, |*r*| = 0.82), whereas no significant difference was observed between P1 and P2. For the eligible VPs from *N* = 200, the distributions of ${CKR}_{Total}$ values also differed significantly (χ^2^ (2, *N* = 456) = 260.5, *p* < 0.001). Post hoc comparisons confirmed significant differences with a large effect size between P1 and P3 (*z* = 14.56, |*r*| = 0.87, *p* < 0.001) as well as between P2 and P3 (*z* = −14.59, *p* < 0.001, |*r|* = 0.88), but no significant difference was observed between P1 and P2. The Mann–Whitney U test revealed that the distributions of ${CKR}_{Total}$ values differed significantly between the two subsets of eligible VPs derived from *N* = 20 and *N* = 200 for P3. The mean rank of ${CKR}_{Total}$ was higher for the eligible VPs from *N* = 20 (78.9) than those from *N* = 200 (53.9) (*z* = −2.03, *p* = 0.043, |*r|* = 0.19), indicating a small effect size. The distributions of the clinical adaptation error rates differed significantly among the three patients for the eligible VPs from *N* = 200 (χ^2^ (2, *N* = 456) = 6.2, *p* = 0.044). Post hoc comparisons revealed a significant difference with a small effect size between P1 and P2 (*z* = −2.45, *p* = 0.043, |*r*| = 0.13), while no significant differences were observed between P1 and P3 or between P2 and P3. The median clinical adaptation error was highest at 2.7% for P2.

## 4 Discussion

In this study, we investigated the clinical adaptation of the Nephroblastoma Oncosimulator to clinical MRI scans, treatment data, and histological profiles from three patients across different clinically assessed risk groups. The main focus of this work was modeling the tumor volume responses to preoperative chemotherapy over time. We evaluated the model’s ability to predict actual tumor volume reductions by quantifying the relative deviations between predicted and observed values (clinical adaptation error) by setting a deviation of 5% as the tolerable adaptation criterion.

The primary goal of the simulations was to determine whether distinct and appropriate joint distributions of the input parameter values could be established for all three risk groups. In particular, we focused on the parameter ${CKR}_{Total}$ that represents the fraction of tumor cells eradicated by chemotherapy and serves as an indicator of treatment effectiveness. We explored the value ranges of ${CKR}_{Total}$ across multiple VPs for each real patient using an optimization algorithm and assessed the corresponding clinical adaptation errors. Additionally, we incorporated histological profiles under consideration of a histology criterion (see Section 2.1) to identify representative simulations runs. We assessed the impact of this consideration on refining suitable parameter distributions and examined the effects of increasing the number of VPs per real patient on the robustness of parameter estimation.

Our results indicate that ${CKR}_{Total}$ varied across the different risk groups and that accurate predictions of tumor volume reductions could be achieved within the defined adaptation criterion. A higher ${CKR}_{Total}$ was associated with greater reduction of the tumor volume, reinforcing its relevance as a key therapeutic descriptor. Patients P1 (low risk) and P2 (intermediate risk) exhibited similar ${CKR}_{Total}$ distributions, whereas P3 (high risk) demonstrated lower ${CKR}_{Total}$ values with reduced variability. This finding aligns with the clinical observations, suggesting that treatment effectiveness generally decreases with increasing patient risk. However, distinguishing between the low and intermediate risk groups remained challenging even when the histology criterion was considered in addition to the adaptation criterion. Although the ${CKR}_{Total}$ distributions differed significantly between the high-risk and other groups, no clear separation was observed between the low- and intermediate-risk patients. The only significant difference between these two groups was observed in the distributions of the clinical adaptation error, albeit with a small effect size. Given that the allowed clinical adaptation error is restricted to 5%, this approach does not appear to hold promise for distinguishing between these two groups. Notably, varying the numbers of VPs (N = 20 vs. N = 200) did not lead to statistically significant differences in the ${CKR}_{Total}$ value distributions regardless of the applied criteria, except for one test for P3 from the high-risk group, where a significant difference was found. However, this difference was also associated with a small effect size, suggesting that the results are stable across different sample sizes for the three patients.

Several modeling assumptions should be considered when interpreting the results of this study. The Nephroblastoma Oncosimulator employs a discretized multiscale approach to model tumor volume development as a response to therapy. The model assumes that tumor volume development follows predefined biological rules and does not explicitly incorporate interactions with the immune system. This may limit model generalizability, especially in cases where immune responses play key roles in therapeutic effectiveness. Additionally, the treatment responses were simulated under the assumption of direct cytotoxic effects of chemotherapy, without accounting for variations in drug penetration or metabolism at the cellular level. This could lead to overestimation of the treatment efficacy. ${CKR}_{Total}$ was selected as the primary parameter to quantify treatment effectiveness because it provides a direct and interpretable measure of tumor volume reduction; however, it does not capture delayed treatment effects. The current model does not integrate the 6-week treatment regimen with additional administration of DOX, which was prescribed for P1. This regimen should be incorporated in addition to the already implemented four-week chemotherapy. Chemotherapeutic resistance mechanisms, such as adaptive resistance and clonal selection, are also not integrated into the model. Another limitation is that the model does not explicitly represent the tumor microenvironment, including factors such as oxygenation, nutrient supply, and extracellular matrix composition. These factors are critical for understanding the full complexity of tumor behaviors and treatment responses. Further refinement to incorporate these factors could enhance the predictive accuracy and relevance of the model.

The MRI data used in this work were characterized by heterogeneous, non-standardized acquisition settings that reflect the real conditions in clinical practice even when protocols are provided. The preprocessing procedure involved manual tumor segmentation, annotation, and resampling, which could introduce bias. However, this bias was quantified to be marginal, as shown in Table 6. The tumor volume calculated using the ellipsoid formula is often an overestimation of the tumor size as the formula assumes a simplified tumor geometry that may not accurately reflect its actual shape. In contrast, manual segmentation accounts for the tumor’s irregular spatial structure by delineating it slice by slice. Although this technique is reliable when validated by experts, its inherent subjectivity and potential for errors should be acknowledged, along with the more time-consuming nature. This underscores the importance of standardization and the potential roles of automated segmentation methods in reducing variability while improving accuracy in clinical assessments.

Although the dataset explored in this work was limited to one patient per risk group, it serves as a foundation for future studies. Additional retrospective clinical and imaging data are already available for further model validation and verification. Expanding the dataset and optimizing the model parameters could help identify more distinct distributions. Since the model operates under specific assumptions, uncertainty quantification is essential for assessing robustness. The adaptation criterion was initially defined on the basis of expert knowledge, which is a common approach in such cases. However, the choice of error metric used to define acceptable deviation in actual tumor reduction is not fixed but rather variable, allowing flexibility in model calibration. The selection of different error metrics may influence the weighting of specific tumor response characteristics, potentially affecting model predictions and their clinical interpretability; these drawbacks also apply to the histology criterion. The influences of different adaptation criteria on model predictions should be further investigated to enable a more nuanced approach to uncertainty quantification and refinement of the clinical adaptation process.

The numbers of VPs per real patient were limited to *N* = 20 and *N* = 200 in the present study as these choices were meant as an initial attempt. Although we did not observe any statistically significant differences between these two VP numbers for the distributions, varying the number of VPs remains relevant. Increasing the number of VPs allows robustness checks of the parameter distributions, ensuring that the findings are not artifacts of small sample sizes. If the distributions remain stable across different VP numbers, it suggests that reliable conclusions can be drawn even with lower numbers of VPs, thereby optimizing the computational efficiency. However, larger VP numbers could still provide more robust parameter estimates to help detect potential model instabilities or sensitivities that may not be evident in smaller samples. This is especially important for ensuring the representativeness of the derived parameter distributions and reduces the risk of the outcomes being influenced by random fluctuations. Higher VP numbers are advantageous when handling more heterogeneous tumor characteristics or complex treatment responses as they allow more accurate representations; this is because subtle differences or trends may not be apparent with smaller sample sizes. The impact of VP number on the model’s performance depends on the underlying variability of the system being modeled. Understanding the point at which increasing VP numbers cease to provide additional insights is crucial for optimizing future simulations. Therefore, systematic evaluations of different VP numbers can help refine the best practices for model application as well as enhance confidence in the stability and generalizability of the results.

We employed a uniform probability distribution for the cell-cycle-related parameters and allowed deviations of up to 50% from the reference values. Since these parameters cannot be directly inferred from patient-specific medical data, a distribution-based variation is a reasonable approach to account for biological uncertainties. The choice of a uniform distribution ensures systematic and broad exploration of the parameter space by preventing bias toward a specific value range. Although a Gaussian distribution might better reflect natural biological variability, precise mean values and standard deviations of the parameters are not sufficiently documented in literature. In the absence of well-established distributions, the uniform distribution was chosen as a pragmatic alternative to ensure that all parameter values were equally likely within the defined range. This approach allows comprehensive representation of biological heterogeneity. Future studies should assess alternative distribution models that might better reflect known biological mechanisms. Evaluating the impacts of different distribution assumptions on model outcomes could enhance the robustness and biological plausibility of the simulations.

One of the advantages of the top–down model is extensibility with new data and sources. Incorporating longitudinal patient data can provide a dynamic view of the tumor responses over multiple time points (Kumar et al., 2012; Jin et al., 2021). Integrating detected genetic and molecular markers like specific mutations or expression profiles (Perotti et al., 2023; Zheng et al., 2023), e.g., for miRNA (Kolokotroni et al., 2024), could help refine the model’s predictive power by accounting for underlying biological differences between tumors (Mahamdallie et al., 2019; Brzezinski et al., 2021). This also includes indications of chemotherapeutic resistance (Choochuen et al., 2024). Analyzing the side effects of chemotherapy and their impacts on treatment is crucial for balancing tumor reduction against the immediate and late adverse effects (Weil et al., 2023). The role of the immune system in tumoral response to chemotherapy could be considered by adding immunological parameters. There is also evidence that the time between start of therapy and surgery could impact preoperative chemotherapy (Meier et al., 2023). Further, exploring radiation treatment and its effects could add value to the model (Wein et al., 2000). Adapting the model to more specific renal tumors, e.g., clear cell sarcoma (Kang et al., 2021), mesoblastic nephroma, and rhabdoid tumor, could enhance the utility of the model. Another crucial aspect that must be considered is the tumor microenvironment, including factors such as hypoxia, vascularization, and stroma interactions, which can influence chemotherapeutic responses (Junttila and de Sauvage, 2013). Literature suggests that multiparametric MRI scans can provide relevant information (Hoffmann et al., 2024). Hence, integrating additional imaging modalities, such as diffusion-weighted imaging, could help improve the assessment of tumor tissue types prior to treatment (Hötker et al., 2021).

Artificial intelligence, machine learning, and related methods, such as deep-learning or neural networks, can assist with the aforementioned tasks. Omics data can be considered as input parameters or histology estimated before surgery (van der Beek et al., 2022; van der Kamp et al., 2023). These technologies are linked to advances in semi-automatic and automatic segmentation of tumor areas (Li et al., 2024). Such methods could help standardize image data provision, reduce preprocessing time, and compensate for data heterogeneity. Comparative studies on MRI-based, manual, and automatic segmentation (Buser et al., 2023) reveal interesting results considering the deviations between these methods. Initial studies show promising results in predicting treatment outcomes using machine learning algorithms (Jin et al., 2021; Li et al., 2023).

Scaling the Nephroblastoma Oncosimulator for clinical use requires further development. Some of the key improvements include increasing the predictive power and versatility. However, these enhancements must be accompanied by further sensitivity analyses to understand the impacts of varying input parameters on model predictions and to quantify the uncertainties of these predictions. Comparing the developed model with other prediction models (Kutikov and Uzzo, 2009; Rickard et al., 2020) could also provide insights into the model performance, capabilities, and limitations. Access to clinical data remains a critical challenge in predictive modeling approaches. To enhance reproducibility, robustness, and generalizability, studies incorporating comprehensive datasets are essential. Simultaneously, a mechanistic multiscale model such as the one presented here differs from purely data-driven artificial-intelligence-based models as it is grounded in various biological, physiological, and biomechanical processes that enable credible predictions even with limited clinical data. Following clinical validation, predictive models have the potential to improve our understanding of cancer biology and contribute to personalized treatment strategies (Karam et al., 2024). They can also serve as clinical decision-support tools, particularly for nephroblastoma, where preoperative chemotherapy regimens still rely on imaging studies reviewed by reference pathologists. Currently, histological data are available only after surgery, but these models could improve decision making by providing insights before surgery and aid in better treatment planning. By leveraging patient-specific data, these models can enhance precision medicine and support more individualized treatment decisions to ultimately improve patient outcomes.

The Nephroblastoma Oncosimulator could also serve as a core component in digital twin frameworks for *in silico* clinical trials. For example, *in silico* trials are often used to evaluate the impacts of dose adjustments or alternative drug combinations in specific risk groups to prioritize the most promising strategies for real-world clinical trials. This could enhance the efficiency of trial design and improve the interpretation of results. Clinician trust and user-friendly interfaces are just as essential as model accuracy for broad clinical adaptation. Hence, the interfaces should be intuitive and seamlessly integrable into existing clinical systems (Elhaddad and Hamam et al., 2024). Additionally, continuous training of healthcare professionals is necessary to ensure effective model utilization. The cost-effectiveness of such models should also be evaluated by weighing the financial investment in model development and maintenance against potential savings from optimized treatment plans.

Interdisciplinary collaborations among oncologists, radiologists, pathologists, bioinformaticians, and data scientists are crucial for continuously refining such models and validating their predictions. Engaging patients and the public in the development process ensures that the models align with stakeholder needs and concerns (Hughes et al., 2023). Although predictive models offer significant potential, their clinical adaptation must be carefully aligned with real-world patient data, as demonstrated in this study. A model’s output must be tailored to the unique characteristics of the patients and risk groups. This adaptability is essential for integrating predictive models into routine clinical practice and transitioning them from theoretical frameworks to actionable decision-support tools. Diverse data sources and multimodality approaches are recommended for such tools (Salvi et al., 2024). By refining such models through the use of larger datasets, improved parameter estimation, and validation studies, predictive simulations can evolve into reliable tools for personalized medicine.

High-fidelity simulations are necessary given that data availability demands are constantly increasing, and this requires access to high-performance hardware or cloud-based computing resources as well as advanced database management systems. To be effective in real-world settings, the models must be seamlessly integrable into clinical information systems and imaging databases. Standardized data formats and automated pipelines for MRI preprocessing and parameter extraction would help reduce clinician workload. Regardless of the technical setup, the models must comply with legal, ethical, and other relevant regulatory principles related to patient consent, data privacy, and data protection. Artificial-intelligence-based approaches pose additional ethical and regulatory challenges (Naik et al., 2022), for which federated learning is emerging as a potential solution (Teo et al., 2024). This approach allows data sharing and model training without compromising patient privacy while addressing the challenges posed by artificial-intelligence-based models.

Our future efforts will focus on validating the proposed model using a larger dataset that is already available. Additionally, parameter estimations will be refined and biological features like immune responses and chemotherapy resistance mechanisms will be considered for integration. These enhancements are expected to further improve the model’s clinical applicability and reliability. In summary, interdisciplinary collaborations, continued model refinement, and addressing the challenges related to data access and ethical considerations will be essential for successfully translating predictive modeling into clinical practice. This ongoing evolution is key to transforming predictive modeling from an academic concept to a practical, clinically relevant, and reliable tool for enhancing patient care.

## Data Availability

The data analyzed in this study are subject to the following licenses/restrictions: The data are available from the authors upon reasonable request. Requests to access these datasets should be directed to Norbert Graf at norbert.graf@uks.eu.
